# Case Report: Barotrauma in COVID-19 Case Series

**DOI:** 10.4269/ajtmh.21-0080

**Published:** 2021-05-18

**Authors:** Ramin Sami, Nafiseh Sereshti

**Affiliations:** 1 Department of Internal Medicine, School of Medicine, Isfahan University of Medical Sciences, Isfahan, Iran;; 2 Isfahan University of Medical Science, Isfahan, Iran

## Abstract

Severe acute respiratory syndrome coronavirus 2 can cause pulmonary complications, such as increased risk of barotrauma (BT), but its prevalence and risk factors are not known. In this case series, the course of BT and its related risk factors were discussed in patients with COVID-19 who were admitted to the intensive care unit. Medical records of the patients with COVID-19 and BT and hospitalized in the intensive care unit for 5 months were extracted. The course of BT and its possible associated risk factors are descriptively presented. Among 103 patients with COVID-19 who were intubated, 13 patients (12.6%) had BT. One patient developed BT before intubation. All patients with BT were male. Half of them developed BT in the first 5 days of intubation. Eight patients (61.53%) had a positive culture for *Klebsiella pneumoniae*. Nine patients (69.9%) died. High positive end-expiratory pressure, coinfection with bacterial pneumonia, and history of lung disease may affect BT incidence. The treatment team should increase their upervision on the ventilator setting, especially in the first week of intubation.

## INTRODUCTION

On March 11, 2020, the World Health Organization (WHO) declared the novel coronavirus, namely severe acute respiratory syndrome coronavirus 2 disease, as a pandemic and global health emergency.^1^ The disease rapidly spread worldwide, causing more than 16 million infections and more than 600,000 reported deaths by July 31, 2020.^2^ This disease has very different manifestations at hospital admission, including fever, cough, and dyspnea. Abdominal pain, myalgia, diarrhea, sore throat, fatigue, and loss of smell are other possible symptoms.^3^ A positive polymerase chain reaction test for the virus on samples from nasopharyngeal swabs is a definitive diagnostic test for COVID-19. This test has high specificity but results in false negatives in some cases.^4^

About 15% of patients with COVID-19 infection develop moderate to severe forms that require hospitalization and respiratory support, and about 5% require intensive care unit (ICU) care and supportive treatments, such as intubation and ventilation.^4,5^ The most common complication in patients with COVID-19 is pneumonia, but other complications may also occur, such as acute respiratory distress syndrome (ARDS), sepsis, septic shock, and multiple organ failure.^6^ The WHO guidelines recommend immediate use of mechanical ventilation in patients with hypoxia, hypercapnia, respiratory fatigue, and disturbed level of consciousness.^6^ Different lung-protective strategies have been expressed in the ventilator settings of ARSD patients to reduce the mortality rate. Mechanical ventilation also has many complications, such as barotrauma (BT), which can increase mortality. High positive end-expiratory pressure (PEEP) with overdistention of the alveoli can lead to alveolar rupture and cause BT.^7^ Barotrauma has many different presentations, including pneumothorax, subcutaneous emphysema, pneumoperitoneum, pneumomediastinum or pneumopericardium, air embolization, tension lung cysts, and hyperinflated left lower lobe.^8^

In this case series, we investigated patients with COVID-19 infection who were facing one of the complications of BT (e.g., pneumothorax, pneumomediastinum, or subcutaneous emphysema) during hospitalization with or without intubation. The predisposing factors, side effects, and mortality rates were discussed.

## METHODS

This retrospective case series was performed at Khorshid Hospital in Isfahan, Iran. This hospital is one of the most important centers for the treatment of patients with COVID-19 in Iran. Patients who were admitted to the ICU from February 22, 2020 to July 5, 2020 and who had evidence of BT were included in the study. This evidence was corroborated by a chest X-ray or computed tomography (CT) scan. The COVID-19 diagnoses of all the patients in the study were confirmed by reverse-transcription polymerase chain reaction on specimens from nasopharyngeal swabs. Intubated patients were treated with the volume mode of ventilator following the ARDS guideline network.^9,10^ In our setting, treatment of the patients included conservative and corticosteroid therapy (based on inflammatory factors and the patient's general condition), and no specific antiviral therapy was used. Demographic characteristics, laboratory tests, respiratory conditions, mechanical ventilator settings, and disease progression and outcomes were extracted from the medical the files of the patients.

## RESULTS

During the 5 months since the outbreak of the coronavirus and the hospitalization of 2,154 patients at Khorshid Hospital, 256 patients (11.9%) were admitted to the ICU; of them, 103 patients (40%) were intubated. Thirteen patients (12.6%) developed BT (one patient was intubated after BT). All patients were male. Some characteristics of the patients are presented in Table 1. Mean age was 57.9 ± 16.7 years in the BT group and 69.3 ± 15.2 years in the other intubated patients. Four patients in the BT group (31%) and 10 patients (11%) in the non-BT group had chronic lung disease. Lactate dehydrogenase levels were 946 ± 733 μ/L in the non-BT group was and 1,027 ± 354 μ/L in the BT group. *Klebsiella pneumonia* was grown in the endotracheal tube culture of eight patients (61.53%). In the endotracheal tube culture of other patients, *Acinetobacter baumannii* and *Staphylococcus aureus* were grown. The mean duration of hospital stay was 27.3 ± 8.2 days in patients with BT and 13.7 ± 8.2 days in other intubated patients.

**Table 1:** Demographic findings of patients who experience barotrauma

Demographics	Values
Age (mean ± SD)	57.9 ± 16.72
Sex, *N* (%)	
Male	13 (100)
Female	0 (0)
Comorbid condition, *N* (%)	
Obesity	2 (15.38)
Overweight	8 (61.53)
DM	3 (23.07)
HTN	2 (15.38)
Chronic lung disease	3 (23.07)
CKD	1 (7.69)
IHD	1 (7.69)
Cancer	1 (7.69)

CKD = chronic kidney disease; DM = diabetes mellitus; HTN = hypertension; IHD = ischemic heart disease.

All the patients had the ARDS criteria, including sudden onset of symptoms within 7 days, bilateral alveolar infiltration, severe progressive hypoxemia in the absence of cardiogenic pulmonary edema symptoms, and a PaO_2_/FiO_2_ ratio of < 300. Based on the severity classification of ARDS, one patient had mild ARDS, four patients (30.76%) had moderate ARDS, and eight patients (61.53%) had severe ARDS. Based on the types of BT, eight patients (61.53%) developed subcutaneous emphysema, and four patients (30.76%) developed multiple type involvement (Table 2). One patient developed pneumothorax before intubation. In intubated patients on the day of BT onset, nine patients (75%) had a PEEP between 5 and 10 cm H_2_O, and three patients (25%) had a PEEP > 10 cm H_2_O. Details of the severity of ARDS and PEEP are shown in Table 2.

**Table 2:** Severity of ARDS and barotraumas finding and associated PEEP

Patient characteristics	*N* (%)	Subcutaneous emphysema	Pneumothorax	Pneumomediastinum ± pneumothorax ± subcutaneous emphysema*	Subcutaneous emphysema + pneumothorax
ARDS severity					
Mild	1 (7.69)	0	0	0	1
Moderate	4 (30.76)	4	0	0	0
Severe	8 (61.53)	4	1	2	1
PEEP					
5–10	9 (75)	7	1	1	0
> 10	3 (25)	1	0	1	1

ARDS = acute respiratory distress syndrome; PEEP = positive end expiratory pressure.

* One patient developed pneumothorax before intubation.

**Figure 1.**
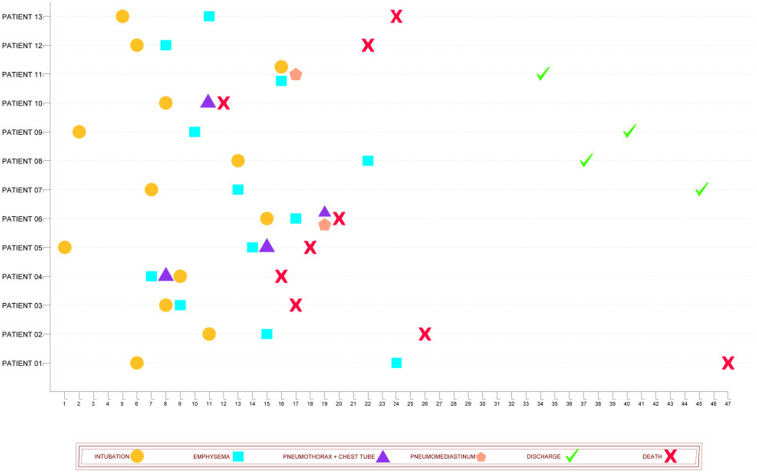
Type of barotrauma and the interval of its occurrence of it in each patient. This figure appears in color at www.ajtmh.org.

In BT patients, the average time between hospitalization and intubation was about 8.2 days, the average time between intubation and BT was about 5.6 days, and the average time between BT to death was about 8.1 days (Table 3). Four patients (30.7%) were discharged, and nine patients died (69.9%). Details of the type of BT and the time of its occurrence are shown in Figure 1.

**Table 3:** nterval (as days) between admission, mechanical ventilation, and barotrauma/death

Patient data	Days			
	0–5	5–10	10–15	>15
Admission to mechanical ventilation				
* N* (%)	3 (23.07)	6 (46.15)	3 (23.07)	1 (7.69)
Mean (range)	8.23 (0–16)			
Mechanical ventilation to Barotraumas				
* N* (%)	6 (50)	4 (33.33)	1 (8.33)	1 (8.33)
Mean (range)	5.6 (0–18)			
Barotraumas to death				
* N* (%)	3 (33.33)	2 (22.22)	3 (33.33)	1 (11.11)
Mean (range)	8.1 (1–23)			
Barotraumas to discharge				
* N* (%)	0	0	0	4 (33.3)
Mean (range)	24 (15–32)			

## DISCUSSION

**Figure 2.**
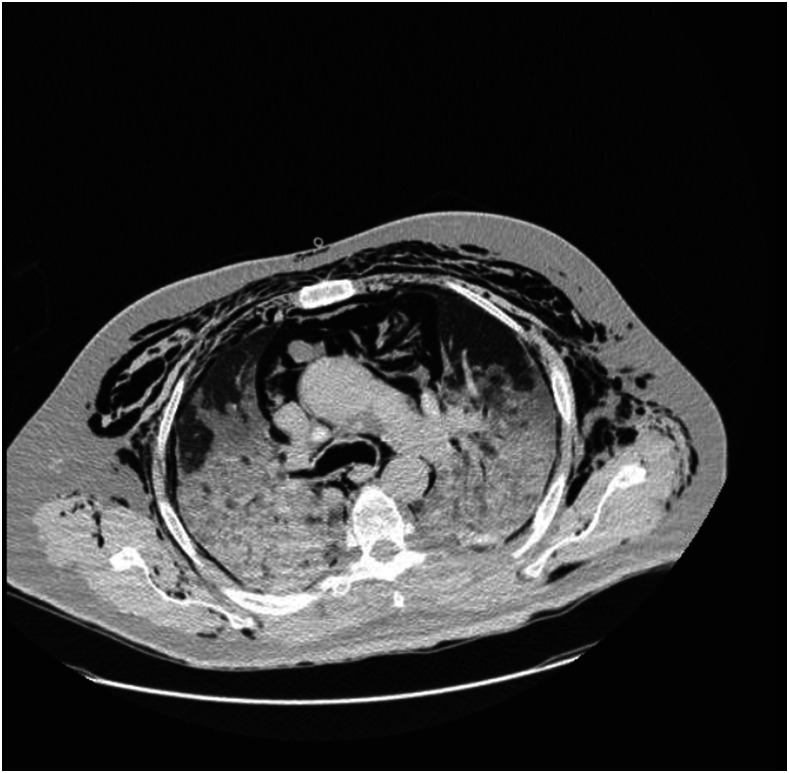
Barotruma events. The patients is a 68-year-old man with history of diabetes who developed bilateral pneumothorax, pneumomediastinum, and subcutaneous emphysema.

Barotrauma caused by mechanical ventilation is a potentially fatal complication reported in 15% of intubated patients.^11,12^ It is more common in patients with an underlying lung disease, such as chronic obstructive pulmonary disease or ARDS.^13^ The knowledge of the complications of the COVID-19 disease has been increasing over the past year. In COVID-19 patients with severe involvement of the lung parenchyma, pulmonary compliance is reduced due to pathological changes such as edema, vascular congestion, and inflammation.^14^ As a result, it is possible that overinflation and high PEEP in such hypoplastic and fibrotic lungs can lead to alveolar rupture and BT.^15^ We show the barotrauma event on a CT scan of one patient in Figure 2.

In the present study, 12.6% of the developed BT. Reports on the prevalence of BT in COVID-19 patients have been reported differently. McGuinness et al.^16^ compared the prevalence of BT in intubated COVID-19 patients and those with ARDS for other reasons. They reported that the prevalence of BT was 15% in patients with COVID-19 and 10% in other patients, and this complication was more common in young people with COVID-19. In a systematic study, Salehi et al.^17^ evaluated CT scans from 3,647 patients with COVID-19, which were published in 37 papers. In this study, pneumothorax was described as an atypical finding in COVID-19 patients, with a low prevalence in CT scan findings. During the past year, cases of BT in COVID-19 patients have been reported in various forms (pneumothorax, pneumomediastinum, and subcutaneous emphysema). Some cases that have attracted the attention of researchers are BT as the first presentation of COVID-19 in the inflammatory phase,^18^ BT in COVID patients without noninvasive or invasive mechanical ventilation,^19^ and BT in patients without underlying lung disease.^20^ Because in some reports BT increased mortality in intubated patients,^20,21^ the authors recommend early imaging to diagnose and treat the complications of COVID-19 pneumonia, such as pneumothorax and subcutaneous emphysema.^22^

All the patients with BT in this study were male. Several reports have shown that more men are infected with COVID-19 than women.^23,24^ More mortality and morbidity have also been reported in men.^25,26^ Similar to the present results, in another case series of BT in COVID-19, the prevalence of BT was higher in men.^11,27^ Bwire et al.^28^ explained the biological differences in the immune system between men and women and stated that women are more resistant to COVID-19 infection than men due to different sex hormones and the higher expression of coronavirus receptors (angiotensin converting enzyme 2) in men. Tracheal culture revealed *Klebsiella pneumonia* in 61% of patients and *Acinetobacter baumannii* in 15% of patients. Bacterial pneumonia superimposed on COVID-19 may predispose patients to BT. Diaz et al.^12^ reported that *Pneumocystis jiroveci* pneumonia is one of the risk factors for BT.

Only one of the patients had BT without mechanical ventilation. Although most studies have reported BT in mechanically ventilated patients, like other viral pneumonia, pneumothorax has been reported in nonintubated COVID-19 patients.^18,19,27^ Udi et al.^11^ compared ventilator settings in patients with and without BT. They reported that there may not be a direct association between BT and mechanical parameters of the ventilator. Although higher airway pressures in mechanical ventilation are expected to be associated with a higher risk of BT, and some studies have reported this,^29^ in most studies, similar to the present study, this association has not been found.^30,31^ It seems that other factors, such as pulmonary compliance, the severity of pneumonitis, patient respiratory effort, coinfection, etc., affect this complication.

In our patients, BT occurred most often (50%) in the first 5 days. Abdallat et al.^32^ reported that the median time between intubation and BT was 3.5 days, and in the case series by Lassence et al.^33^ it was 4 days. Therefore, it is recommended that more attention be paid to this complication at this time.

## CONCLUSION

In this article, BT was investigated as a less-reported complication of COVID-19 infection. It seems that middle-aged men are more susceptible to this complication, especially in the first 5 days after intubation. According to the present findings, a high PEEP in mechanical ventilation, coinfection with bacterial pneumonia, and a history of chronic lung disease may have considerable effects on BT incidence. This complication leads to longer hospital stays for patients, but it does not necessarily increase mortality. It is recommended that low pressure be used to adjust the ventilator, the ventilator be adjusted on a daily basis according to the patient's condition, and examination for subcutaneous emphysema be performed, especially in the first week of intubation.

### Limitation.

Because this is a retrospective study and CT scans were not performed on all the intubated patients, mild cases of BT were not diagnosed. The present data are related to overt BT. This is a case series and the demographic data, characteristics, and outcomes of the two groups could not be compared.
